# Supplementary material for: Trematode infection in ruminants and diversity of snail hosts across three agro-ecological zones in Ethiopia
Source: BMC Vet Res. 2024 May 13;20:197. doi: 10.1186/s12917-024-04049-0 (PMC11089747; doi:10.1186/s12917-024-04049-0)
Supplement: Supplementary file 1 — Supplementary Material 1 [file 12917_2024_4049_MOESM1_ESM.docx]

**Trematode Infection in Ruminants and diversity of Snail Hosts across Three Agro-Ecological zones in Ethiopia**

Bekele Megersa^1^, Bilisuma Hussein^2^, Jemila Shemsu^1^, Redeat Kassahun^1^, Olana Merera^1^, Nebyou Moje^1^, Bedaso Mammo Edao^1,3^ Hika waktole^1^, Hagos Ashenafi^4^, Dinka Ayana^1^

# Authors Affiliation:

^1^College of Veterinary Medicine and Agriculture, Addis Ababa University, Bishoftu Ethiopia

^2^College of Veterinary Medicine, Hawassa University, Hawassa Ethiopia

^4^Aklilu Lemma Institute of Pathobiology, Addis Ababa University, Addis Ababa Ethiopia

^3^Department of Pathobiology, University of Illinois, USA

# Corresponding author email: [bekelebati@gmail.com](mailto:bekelebati@gmail.com)

**Abstract**

The occurrence of trematodes among ruminants and their snail vectors is a major concern across various agro-ecological regions of Ethiopia. Trematodes pose significant threats to animals, causing considerable economic losses and impacting public health. In this study, we have investigated 784 ruminant fecal samples, and 520 abattoir samples, alongside the collection and identification of snail vectors from various agro-ecological regions. Fecal examinations revealed Fasciola, Paramphistomum and Schistosoma species infected 20.5% (95% CI: 17.6, 23.8), 11.7% (95% CI: 9.6, 14.2), and 6.3% (95% CI: 4.1, 9.1) of the animals, respectively. The overall prevalence of trematodes among ruminants was 28.8% (95% CI: 25.7, 32.1%), with 6.0% (95% CI: 4.3, 7.7) showing mixed infections. Fasciola was more prevalent in Asela (26%) compared to Batu (19%) and Hawassa (11.5%), while a higher proportion of animals in Batu were infected with Paramphistomum. Schistosoma eggs were detected only in Batu (12.5%), but not in other areas. Sheep and cattle exhibited higher infection rates with Fasciola, Paramphistoma, and Schistosoma compared to goats. Significant associations were observed between trematode infections and risk factors including agro-ecology, animal species, body condition score, and deworming practices. About 20.8% and 22.7% of the slaughtered animals harbored Fasciola and Paramphistomum flukes, respectively, with a higher prevalence in Asela and Hawassa abattoirs compared to Batu abattoir. Additionally, a total of 278 snails were collected from the study areas and identified as *lymnae natalensis, lymnae trancatula, Biomphalaria pffiferi, Biomphlaria sudanica,* and *Bulinus globosus*. In conclusion, the study highlights the widespread occurrence of trematode infections, emphasizing the need for feasible control measures to mitigate their economic and public health impacts.

**Keywords:** Trematode Infections, Ruminants, Snail Vectors, Agro-ecology, Ethiopia

1. **Introduction**

The large population of ruminants in the country plays a vital role in the livelihoods, food security, and socio-economic dynamics of rural communities (1). Ruminants, such as cattle, sheep, and goats predominantly managed under extensive grazing systems, are consistently exposed to parasitic infections throughout the year, rendering them vulnerable to a heavy burden of disease(2) In particular, trematode infections among ruminants and their associated snail vectors are a major concern in various agro-ecological regions of Ethiopia. More importantly, Ethiopia's diverse agro-ecological zones, ranging from highlands to lowlands, offer varied habitats for the survival and transmission of trematode parasites and their intermediate hosts, freshwater snails.

The epidemiology of trematode infections is influenced by various factors, including environmental conditions, host susceptibility, and the presence of intermediate hosts. Wide range of environmental factors (e.g. climate, altitude, rainfall patterns, water bodies), and agricultural activities thus affect the occurrence of trematode infections, leading to spatial and temporal variations in disease prevalence and distribution (3,4). Prevalence of trematode infection varies considerably by macro and microclimatic conditions that facilitate the survival and interactions with these intermediate hosts. Thus, snails play a crucial role in the occurrence and the geographic distribution of trematode species (5). Hence, understanding the distribution patterns and prevalence of major trematode infections in ruminants, as well as the diversity of their vector species across various agro-ecological zones, is so crucial for developing feasible control measures.

Trematodes, belonging to the Digenea subclass, constitute a diverse group of parasites having capability to infect a wide range of invertebrate and vertebrate hosts. Consequently, they exhibit a complex life cycle usually involving multiple hosts including intermediate snail vectors and final vertebrate hosts including human, mammals, fish, and birds (3). In the final host, they can affect various organs such as the digestive tract, lungs, liver, and vascular system. The infections can contribute significantly to organ damage and production losses in the livestock in addition to posing risk to public health (4,6).

Trematode infection is highly prevalent in livestock across tropical regions and is recognized as the most significant helminthic parasite affecting livestock production, resulting in considerable economic losses (7,8). In particular, fasciolosis caused by liver flukes of the genus Fasciola, in particular *F. hepatica and F. gigantica*, is characterized by hepatic damage reduced productivity, and mortality in severe cases (9,10), and economic losses resulted from organ condemnation (11,12). Cwiklinski and colleagues (13) have estimated the global economic burdens associated with fasciola infection in ruminants to exceed USD 3 billion. Paramphistomosis predominantly affects the digestive tract by causing inflammation and damage to rumen mucosa, papillary body and villi atrophies, leading to digestive disturbances, decreased feed conversion and weight gain, besides condemnation of the rumen (9). The parasites have a broad range of final hosts, including both domestic and wild animals, and they exhibit a wide geographical distribution, with growing emergence observed in European countries (14). Likewise, various Schistosoma species, including *S. bovis, S. mattheei, and S. japonicum*, have also the capability to infect ruminants, posing a dual threat to both animal and human health. Indeed, the occurrence of human Schistosoma species, such as *S. haematobium*, in cattle, as well as the existence of interspecies hybridization between *S. bovis and S. haematobium*, suggests the increasing public health risks associated with animal schistosomiasis where animals may serve as reservoirs for human infections (15).

Human trematode infections typically occur through ingestion of contaminated water and food, or contact with intermediate hosts and being regarded as emerging public health threat (16). Indeed, human-infecting trematodes encompass a diverse range of genera; approximately 60 of them are recognized to cause disease in human (3). They are classified based on their predilection site within the definitive hosts such as blood, liver, lung and intestinal flukes. Among these, Fasciola, Schistosoma, Opisthorchis, and Paragonimus species commonly infect people. Schistosoma in particular, is well known for causing schistosomiasis, a widespread disease impacting millions of people worldwide (17). Fasciolosis is also an emerging parasitic zoonosis that has been regarded as the most neglected tropical diseases, significantly affecting human health. Cases of human fascioliasis have been reported from 12 African countries (18), including Ethiopia (19–21). World Health organization estimated about 2.4 million people are infected globally, and over 180 million are at risk of infection in several countries, particularly where ruminants are extensively reared (22). Schistosomiasis is indeed a neglected tropical disease, with an estimated 779 million people at risk of infection, and about 250 million currently infected, most of whom are Africans (17). These parasites pose high health burden, particularly to rural population with limited access to healthcare, leading to severe health complications, including organ damage, malnutrition and hindered cognitive development in children.

While a number of studies have explored the prevalence of specific trematode species in different animal species in Ethiopia (14,23), there has been a lack of research specifically focusing on major trematodes across all ruminant species in various agro-ecology. With the exception of few studies (24–26) literature on the occurrence of major trematodes in bovines, small ruminants and associated snail vectors is limited. Thus, this study investigated the epidemiology of trematode infections in ruminants and characterized the associated snail vectors in selected agro-ecological zones in Ethiopia. By illustrating the prevalence, distribution and associated risk factors, this research provides evidence for necessary interventions aimed at minimizing the burden of these parasitic diseases on livestock and public health.

# 2. MATERIALS AND METHODS

## Description of the Study Area

The study was conducted between November 2021 and April 2022 in three areas: Asela, Hawassa, and Batu, which represent different agro-ecologies (Fig. 1). Asela, situated in the central highlands of Ethiopia, has a latitude and longitude of 7°57′N and 39°7′E, and an elevation of 2,430 meters. The area exhibits a highland agro-ecology characterized by high average annual rainfall of 1200 mm, and an annual temperature range of 5°C to 28°C. Batu and Hawassa are located within the Great Rift Valley, which extends across East Africa. Both towns are situated on the shores Lake Hawassa and Lake Ziway (Batu), respectively. Hawassa is located at 38°29′E and 7°05′N, approximately 275 kilometers south of Addis Ababa, with an elevation of 1790 meters above sea level. The agro-ecology of the Hawassa conform to a mid-altitude climate, characterized by an average annual rainfall of 960 mm and average annual minimum and maximum temperatures of 12°C and 28°C, respectively. Batu town is found at 163 kilometers south of Addis Ababa, with latitude of 38°43′E and a longitude of 7°56′N and an elevation of 1643 meters above sea level. The area has an average annual rainfall of 930 mm and average minimum and maximum temperatures of 10°C and 30°C, respectively, which may categorize it as a lowland agro-ecology. All three towns have municipal abattoirs dedicated to slaughtering animals and supplying meat to the local residents. Various forms of livestock production, including semi-intensive and predominantly extensive production systems, are commonly practiced in the urban and peri-urban, and rural areas of these regions (27–29).


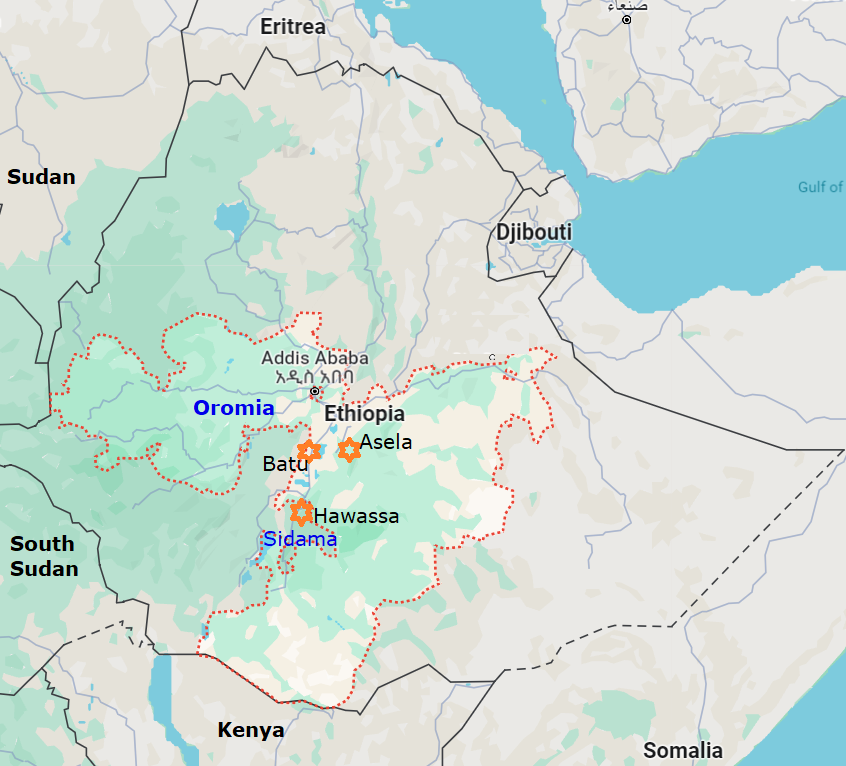


Figure 1. Map of Ethiopia showing the study areas: Batu and Asela from Oromia region, and Hawassa from Sidama region (adapted from Google map).

## Sampling Methods

We applied a simple random sampling method to select the study farms and animals for fecal examination. About 100 or more registered cattle herds (farms) are estimated to exist in urban settings of the areas. Additionally, a higher number of unregistered cattle herds or small ruminant flocks are anticipated to be herded in suburban areas (27–29). Accordingly, lists of cattle herds (farm owners) were obtained from the agriculture offices of each town, from which farms were randomly selected for inclusion in the study. Subsequently, animals from the selected farms were sampled using a lottery system, with each animal having a 50% chance of being selected from a target herd until the required sample size reached. For unregistered herds or small ruminant flocks, owners of encountered animals were contacted to obtain their consent, following which their animals were sampled for fecal collections.

Similarly, for cattle slaughtered in each abattoir, a systematic random sampling approach was employed, considering an estimated number of approximately 20 to 50 cattle slaughtered per day at each municipal abattoir. From a total of slaughtered animals per day, 10 animals were randomly selected for investigation (30). Given the irregular and smaller numbers of sheep or goats slaughtered at abattoirs, all small ruminants slaughtered were investigated for the presence of flukes.

## Study Settings

A cross-sectional study was undertaken involving three ruminant species of both sexes, sampled from designated study areas. The study population comprised domestic ruminants commonly found in these areas, including cattle, sheep, and goats, predominantly of local breeds. Approximately 29% of the cattle were cross-breeds, managed semi-intensively. The animals were predominantly kept under extensive farming practices, freely grazing in fields. Small ruminants, such as sheep and goats, were mainly reared in urban areas with free-roaming practices and extensively in suburban regions. For postmortem examination aimed at detecting flukes, a cross-sectional study was implemented at municipal abattoirs found in the three towns. Additionally, snail collections and subsequent identification were conducted in the study areas using identification keys.

## Sample Size Determination

To estimate the sample size (n), we used the Cochran formula(31) for unknown population size but known expected proportion (p), considering a 95% confidence interval (Z) and a 5% desired precision (d):

$$n=\frac{p*\left( 1-p \right)*\left( Z \right)^{2}}{\left( d \right)^{2}}$$

The study areas were grouped into two populations: Hawassa and Batu in one population, while Asela was regarded as the second population due to its different agro-ecology and location. Hawassa and Batu were considered as one study population as both areas are situated in the Rift Valley region and are located along the shores of Rift Valley lakes, contributing to their similarity in some environmental and ecological features. We considered the overall trematode prevalence of 61% reported (25) from lowland area to estimate the number of animals (cattle) for fecal sampled from Hawassa and Batu which gives 366 animals. For Asela area, we estimated a total of 383 animals, considering the expected prevalence of 48% reported for the overall prevalence of fasciolosis in Bale highland areas with similar agro-ecology (32). Similarly, a comparable number of slaughtered animals were considered for the abattoir postmortem inspection and detection of flukes. The intended sample size for fecal examination was adequately met, with 782 animals underwent coproscopic examination. Among these, 400 samples were collected from Hawassa and Batu, and 382 were obtained from Asela (with one sample lost during processing). While a significant number of animals (n=520) were subjected to postmortem inspection, this figure fell below the intended targets due to the predominate practices of backyard slaughtering among small ruminants.

**Sample collection and processing**

Before sample collections, each animal selected for study were identified by providing separate identification number which was used in both fecal examination and abattoir for postmortem examination. Age, sex, breed, body condition score were recorded upon ante mortem examination. Body condition score (BCS) is a qualitative measure, also on numerical scales of 1 – 5 based on its body fat reserves and muscle mass (by observations of the ribs, spine, hip and tail regions), to assess the health condition and nutritional status of the animals.

In case of fecal examination, age, sex, breed, species, deworming history, origin were recorded. Fresh fecal samples were collected directly from the rectum of each animal using sterile rectal plastic glove. Each sample was carefully kept in air tight clean plastic container containing 10% formalin, and labeled with the animal's identification details. Various factors related to the animals were recorded during the sampling process. This included the body condition score, determined according to the method outlined (33). Additionally, information such as the sex and age of the animal, determined by dentation following protocols (34) respectively, were recorded. Other recorded factors included the breed of the animal, its deworming history and management system.

### **Coprological examination**

Fecal samples were examined for the presence of trematode eggs at the Veterinary Parasitology laboratories of Addis Ababa University and Hawassa University. Samples that were not immediately examined were stored in a refrigerator at a temperature of 4°C to maintain their integrity. Upon examination, approximately 3 grams of fecal sample was weighed and added to a beaker containing 42 ml of 0.1% NaOH solution and thoroughly stirred. The suspension was then filtered through a sieve or strainer and allowed to stand for 5 min to allow the trematode eggs to settle at the bottom of the tube. The sedimentation procedure is repeated until the fecal debris was adequately removed. Then sediment was recovered into a test tube and re-suspended in about 5 ml tap water, and a drop of methylene blue was added and allowed to stand for 5 min to stain the debris. Subsequently, all the materials were transferred into a Petri dish and examined under low power objectives (10x and 40x magnification). Trematode egg counts were performed by moving the Petri dish in such a way that every field was examined. Yellowish brown color of Fasciola spp. eggs was used to differentiate them from those of paramphistomum. Schistosoma species were identified by their oval egg shape, size and sites of terminal spines. Identification of trematode eggs was conducted based on established keys and descriptions found in various literature sources (35–37).

**Postmortem examination**

Organs from animals slaughtered at municipal abattoirs were examined for the presence of flukes in their predilection sites. Each animal included in the study was assigned a unique identification number, and variables such as origin, breed, sex, age, and body condition scores were recorded during the ante-mortem examination. Subsequently, each selected animal underwent postmortem inspection, with particular attention paid to organs where flukes typically localize, including the liver, lung, mesenteric vein, rumen, and reticulum. The presence of trematode parasites was determined through careful inspection, involving visualization, incision, palpation and further visual examination of the organs.

**Snail species identification**

Snails were collected using the gloved hand-picking method from representative water bodies, wet and marshy areas in the study areas (Asela, Batu and Hawassa). The mollusks were placed in plastic bags along with fresh water algaeand aerated water to maintain their habitat conditions. Then 70% ethylalcohol was added to preserve the specimens (38). Subsequently, the samples were transported to the laboratory for identification. Identification keys, including parameters such as shell size, number of whorls, columella, shell shape, surface characteristics, colours and patterns were employed for this purpose (38,39).

## Data management and analysis

All data were entered into a Microsoft Excel spreadsheet, meticulously checked for any errors, then processed and analyzed using Stata version 14 (Stata Corp. College Station, Texas, 77845 USA). Independent variables including sex, age, breed, species, body condition score and deworming history were analyzed for the association with trematode status on the basis of coprological and postmortem results. Summary statistics like frequency distributions and percentages were employed to descriptively present the data. Trematode detections by coprological and postmortem methods were compared using two proportions comparison test as indicator of diagnostic efficiency. Furthermore, firthlogit model was performed to investigate the association between risk factors and the presence of trematode eggs in fecal samples, as well as the detection of flukes to accommodate zero observations. Data regarding the overall infections of the animals by the three trematode genera were generated by summing the infection status of each flukes. Then, multivariable negative binomial regression was applied to analyze factors associated with the overall trematode infections (count variable ranging from 0 to 3) using incidence risk ratio (IRR) to measure the strength of association.

**3. RESULTS**

**Prevalence of Trematodes in Ruminants**

Out of the 784 ruminants subjected to fecal examination, 20.5%, 11.7%, and 6.3% were found to be infected with Fasciola, Paramphistomum, and Schistosoma species, respectively. Overall, 226 out of the 784 ruminants investigated were found to be infected with trematodes, resulting in a global prevalence of 28.8%. Mixed infections involving two or all the three flukes were also observed in 6.0% of the animals. Fasciola exhibited a higher prevalence in Asela (26%) compared to Batu (19%) and Hawassa (11.5%), while a higher proportion of animals in Batu were infected with Paramphistomum (17.5%) and Schistosoma (12.5) compared to other areas (Table 1). No Schistosoma eggs were detected during fecal examination in the Hawassa and Asela areas. Goats showed a lower prevalence of infection with Fasciola, Paramphistomum, and Schistosoma compared to sheep and cattle.

Table 1: Prevalence of Trematodes in ruminants based on fecal examination

| Variables | Sample | Fasciola | | Paramphistomum | | Schistosoma* | |
| --- | --- | --- | --- | --- | --- | --- | --- |
|  |  | Positive | % (95% CL) | positive | % (95% CL) | positive | % (95% CL) |
| **Areas** |  |  |  |  |  |  |  |
| Batu (LL) | 200 | 38 | 19.0 (13.8, 25.1) | 35 | 17.5 (12.5, 23.5) | 25 | 12.5 (8.3, 17.9) |
| Hawassa ML | 200 | 23 | 11.5 (7.4, 16.8) | 21 | 10.5 (6.6, 15.6) | 0 | 0 |
| Asela (HL) | 384 | 100 | 26.0 (20.7, 30.7) | 36 | 9.4 (6.7, 12.7) | 0 | 0 |
| **Ruminant** |  |  |  |  |  |  |  |
| Goats | 112 | 12 | 10.7 (5.7, 18.0) | 4 | 3.6 (1.0, 8.9) | 1 | 0.9 (0.0003, 6.2) |
| Sheep | 220 | 54 | 24.5 (19.0, 30.8) | 17 | 7.7 (4.6, 12.1) | 8 | 11.0 (4.9, 20.0) |
| Cattle | 452 | 95 | 21.0 (17.4, 25.1) | 71 | 15.7 (12.5, 19.4) | 16 | 6.7 (3.9, 10.6) |
| Total | 784 | 161 | 20.5 (17.6, 23.8) | 92 | 11.7 (9.6, 14.2) | 25 | 6.3 (4.1, 9.1) |

LL: lowland, ML: midland, HL, highland

An abattoir survey conducted on slaughtered animals (n=520) revealed that 20.8% and 22.7% of the ruminants were infected with Fasciola and Paramphistomum flukes respectively. These flukes were found to be more prevalent in animals slaughtered at Asela and Hawassa abattoirs compared to those from Batu (Table 2). Despite attempts to detect adult Schistosoma flukes from the intestine and mesenteric vein, no parasites were found in their respective predilection sites.

Table 2: Prevalence of Fasciola and Paramphistomum in slaughtered animals

| Variables | Sample | Fasciola | | Paramphstomum | | Mixed | |
| --- | --- | --- | --- | --- | --- | --- | --- |
|  |  | Positive | % | Positive | % | positive | % |
| Study area |  |  |  |  |  |  |  |
| Batu (LL) | 292 | 33 | 11.3 | 45 | 15.4 | 12 | 4.1 |
| Hawassa (ML) | 108 | 20 | 18.5 | 41 | 38.0 | 11 | 10.2 |
| Asela (HL) | 120 | 55 | 45.8 | 32 | 26.7 | 13 | 10.8 |
| Ruminants |  |  |  |  |  |  |  |
| Small ruminant | 119 | 33 | 27.7 | 18 | 15.1 | 10 | 8.4 |
| Cattle | 401 | 75 | 18.7 | 100 | 24.9 | 26 | 6.5 |
| Total | 520 | 108 | 20.8 | 118 | 22.7 | 36 | 6.9 |

## LL: lowland, ML: midland, HL, highland

Table 3 presents a comparison of the detection of flukes using postmortem and fecal sample examination methods. While direct comparison between abattoir and fecal examinations is challenging given the differences in the animal assessed, it is noteworthy that the detection rates of Fasciola and Paramphistomum were higher by post-mortem inspection compared to fecal sample examination in the Hawassa and Asela areas. In the contrary, in the Batu area, fecal examination resulted in higher detection rates of Fasciola and Paramphistomum compared to post-mortem examination. Consequently, there is no significant disparity between the two detection methods. It is important to consider that animals slaughtered at municipal abattoirs may originate from various areas with different agro-ecological conditions, potentially influencing the prevalence and distribution of trematode infections.

Table 3. Comparison of Fasciola and Paramphistomum prevalence values from post-mortem inspection and fecal examinations

| Location |  | Post-mortem inspection |  | Fecal | P-value |
| --- | --- | --- | --- | --- | --- |
|  |  |  |  |  |  |
| ***Fasciola*** |  |  |  |  |  |
| Batu (LL) |  | 11.3% |  | 19.0% | 0.017 |
| Hawassa (ML) |  | 18.5% |  | 11.5% | 0.091 |
| Asela (HL) |  | 45.8% |  | 26.0% | 0.000 |
| Total |  | 20.8% |  | 20.5% | 0.896 |
| ***Paramphistomum*** |  |  |  |  |  |
| Batu (LL) |  | 15.4% |  | 17.5% | 0.535 |
| Hawassa (ML) |  | 38.0% |  | 10.5% | 0.000 |
| Asela (HL) |  | 26.7% |  | 9.4% | 0.000 |
| Total |  | 22.7% |  | 11.7% | 0.000 |

## LL: lowland, ML: midland, HL, highland

## Risk factors associated with occurrence of trematodes based on fecal examination

Significant associations were observed between trematode infections and various factors, including study location (agro-ecology), animal species, body condition score, and deworming practices (Table 4). Sheep had a higher prevalence of Fasciola and Schistosoma, whereas Paramphistomum was more prevalent in cattle compared to other ruminants. Animals with poor body condition scores showed significantly higher prevalence of trematode infections compared to those in good condition. Recent anthelmintic treatments were found to significantly reduce the prevalence of Fasciola and Schistosoma, while semi-intensive management had a notable effect on the prevalence of Paramphistomum. Trematode occurrence was more prevalent in local breeds and animals kept under extensive production systems. Additionally, Paramphistomum was found to be more prevalent in male and adult animals compared to female and you animals, respectively.

Ruminants in the Asela area were found to be more susceptible to Fasciola infections compared to those from other areas, while Paramphistomum and Schistosoma infections were higher in ruminants from the Batu area.

Table 4. Factors associated Trematode infections in ruminants based on fecal examination

| Variables | Levels | Sample | Fasciola (%) | P-Value | Paramphistomum (%) | P-Value | Schistosoma **(%) | P-Value |
| --- | --- | --- | --- | --- | --- | --- | --- | --- |
| Species | Goats | 112 | 10.7 |  | 3.6 |  | 1.1 | 0.035 |
|  | Sheep | 220 | 24.5 |  | 7.7 |  | 11.0 |  |
|  | Cattle | 452 | 21.0 | 0.012 | 15.7 | 0.000 | 6.7 |  |
| Area | Batu (LL) | 200 | 19.0 |  | 17.5 |  | 12.5 | 0.000 |
|  | Hawassa (ML) | 200 | 11.5 |  | 10.5 |  | 0.0 |  |
|  | Asela (HL) | 384 | 26.0 | 0.000 | 9.4 | 0.012 | na |  |
| Sex | Female | 359 | 20.1 |  | 8.9 |  | 4.7 | 0.362 |
|  | Male | 425 | 20.9 | 0.760 | 14.1 | 0.024 | 7.0 |  |
| Age | Young | 320 | 18.1 |  | 6.9 |  | 7.1 | 0.534 |
|  | Adult | 464 | 22.2 | 0.165 | 15.1 | 0.000 | 5.6 |  |
| BCS | Poor | 324 | 28.1 |  | 16.0 |  | 12.4 |  |
|  | Good | 460 | 15.2 | 0.000 | 8.7 | 0.002 | 3.3 | 0.000 |
| Breed* | Local | 320 | 22.2 |  | 19.7 |  | 6.0 | 0.248 |
|  | Cross-breed | 132 | 18.2 | 0.324 | 6.1 | 0.000 | 13.3 |  |
| Mgt* | Extensive | 368 | 21.5 |  | 17.7 |  | 6.0 | 0.248 |
|  | Semi-intensive | 84 | 19.0 | 0.632 | 7.1 | 0.017 | 13.3 |  |
| Deworm | No | 684 | 21.6 |  | 12.1 |  | 7.0 | 0.081 |
|  | Yes | 100 | 13.0 | 0.045 | 9.0 | 0.363 | 0.0 |  |

* Mgt (Management) and breed data were mainly for cattle, **Schistosoma prevalence (n=400) does not include Asela data, LL: lowland, ML: midland, HL, highland

We applied multivariable negative binomial regression (Table 5) to analyze the factors affecting the overall infections by three trematodes. Our findings revealed the risk of infections had significant association with exposure variables, including the geographical area (agro-ecology), ruminant species, body condition score, and deworming practices.

Increase in altitude from lowland (Batu) to midland (Hawassa) and highland (Asela) areas significantly decreased the risk of trematode infections in ruminants by factor of 0.47 and 0.74 times respectively. Moreover, sheep and cattle exhibited significantly higher rates of trematode infection, with incidence rate ratios (IRRs) 2.2 and 2.5 times greater than that of goats, respectively. Animals with good body condition scores had a lower incidence risk ratio (IRR = 0.64) of trematode infections compared to those in poor condition. Additionally, implementing a semi-intensive production system and deworming practices significantly reduced the risk of infection by 0.63 and 0.56 times, respectively.

Table 5. Factors associated with Trematode infections in ruminants

| Variables | Levels | IRR | P-value |
| --- | --- | --- | --- |
| Area | Batu (lowland) | Ref |  |
|  | Hawassa (midland) | 0.47 | 0.000 |
|  | Asela (highland) | 0.74 | 0.045 |
| Species | Goats | ref |  |
|  | Sheep | 2.19 | 0.004 |
|  | Cattle | 2.53 | 0.000 |
| Sex | Female | Ref |  |
|  | Male | 1.20 | 0.159 |
| Age | Young | Ref |  |
|  | Adult | 1.28 | 0.061 |
| Body condition score | Poor | Ref |  |
|  | Good | 0.64 | 0.000 |
| Management | Extensive | Ref |  |
|  | Semi-intensive | 0.63 | 0.041 |
|  | No | Ref |  |
| Deworming | Yes | 0.56 | 0.012 |
| _cons |  | 0.23 | 0.000 |

IRR: incidence rate ratio

## Factors associated with trematode infections based on postmortem examination

The study findings demonstrated a significant association between the occurrence of Fasciola and various factors including agro-ecology, body condition score, age, and species of animals (Table 6). Additionally, the study revealed that the occurrence of Paramphistomum was linked to the location, species, sex and age of the animals, with higher prevalence observed in the Hawassa area and in male and adult animals. Higher prevalence was also noted in cattle compared to small ruminants. But there was no observed association between body condition score and Paramphistomum infections. The presence of mixed infections by trematode species was observed to be associated with the body condition of ruminants (data not displayed).

Table 6. Factors affecting Fasciola and Paramphistomum occurrence in slaughtered animals

| Variables | Levels | Sample | Fasciola  (%) | P-Value | Paramphistomum (%) | P-Value |
| --- | --- | --- | --- | --- | --- | --- |
| Species | Small ruminant | 119 | 27.7 |  | 15.1 |  |
|  | Cattle | 401 | 18.7 | 0.033 | 24.9 | 0.025 |
| Location | Batu (LL) | 292 | 11.3 |  | 15.4 |  |
|  | Hawassa (ML) | 108 | 18.5 |  | 38.0 |  |
|  | Asela (HL) | 120 | 45.8 | 0.000 | 26.7 | 0.000 |
| Sex | Female | 105 | 26.7 |  | 10.5 |  |
|  | Male | 415 | 19.3 | 0.095 | 25.8 | 0.001 |
| Age | Young | 136 | 14.7 |  | 16.2 |  |
|  | Adult | 384 | 22.9 | 0.043 | 25.0 | 0.035 |
| BCS | Poor | 91 | 40.7 |  | 27.5 |  |
|  | Good | 429 | 16.6 | 0.000 | 21.7 | 0.231 |

## LL: lowland, ML: midland, HL, highland

## Malacological study

Snail samples were collected from study areas (water body and surrounding wet surfaces of study areas) were found to belong to three genera. A total of 278 snails (116 from Asela, 89 from Batu and 73 from Hawassa) were identified based on identification keys described by researchers (39,40). Accordingly, the snails were identified to be *lymnae natalensis, lymnae trancatula, Biomphalaria pffiferi, Biomphlaria sudanica and Bulinus globosus* (Table 7).

Table 7: Snail species and their occurrence in the study areas

| Snail species | Proportion of Snail species (%) found in the study sites | | |
| --- | --- | --- | --- |
|  | Batu | Hawassa | Asela |
| *Lymnae natalensis* | 12 (16.4) | 16 (17.9) |  |
| *Lymnae trancatula* | 8 (10.9) | 12 (13.5) | 116 (100) |
| *Biomphalaria pfeifferi* | 11 (15.1) | 13 (14.6) |  |
| *Bulinus globosus* | 9 (12.3) | 9 (10.1) |  |
| *Biomphlaria sudanica* | 13 (17.8) | 8 (8.9) |  |

# 4. DISCUSSION

The present study investigated the prevalence of trematode infections among ruminants and the diversity of snail vectors across lowland, mid-altitude and highland agro-ecological areas. The findings revealed an overall prevalence of ruminant trematodes, determined through coprological examination at 35.5%. This figure surpassed a previous finding from Wolaita (41), which reported a prevalence of 26.04%. However, the findings from the current research shows a lower prevalence compared to previous accounts where 47 % - 61%, were reported from Bahir Dar, Andassa and northeastern of Amhara region (21, 37, 20). The observed variations in the occurrence of trematode across the studies may be attributed to differences in study seasons, agro-ecological conditions across the study areas, animal management practices, and the availability of suitable habitat for the snail intermediate host (3,4).

In this study, Fasciola (20.5%) showed the highest occurrence, followed by Paramphistomum (11.7%), with Schistosoma being the least prevalent (6.3%). A similar pattern of trematode occurrence was also noted in another study (41). However, our findings contradict the observations of (24) and (25), where a higher prevalence of Paramphistomum compared to Fasciola and Schistosoma was reported. These two studies were conducted in lowland agro-ecology where suitable vectors for Paramphistomum are more abundant, potentially leading to an increased prevalence of rumen fluke. Generally, trematode prevalence varies considerably based on the abundance of intermediate hosts, as well as favourable ecological and climatic factors of the study areas. In our study, we considered different agro-ecological conditions, and Fasciola prevalence was notably high in the highland area of Asela, which may explain its relatively higher prevalence.

At species level, our study findings showed that Fasciola was prevalent in 24.5%, 10.7% and 21% of sheep, goats, and cattle, respectively. The overall prevalence of ovine fasciolosis closely agreed with previous research conducted in various regions of Ethiopia, such as 29.2% in Butajira and Gilgel Gibe (21), albeit lower figures reported by others (43), (44) and (26). Similar to a previous finding (21), our study indicated a low prevalence of caprine fasciolosis in comparison with other ruminants. This could be attributed to the browsing behavior of goats, leading to less consumption of grass in marshy areas. Additionally, a disproportional sample size occurred due to fewer caprine species in our study population. The prevalence of Fasciolosis in Asela (26%) outweighed that in Batu (19%) and Hawassa (11.5%). Asela possesses shallow water bodies and wet areas that provide favorable conditions for the survival and reproduction of the intermediate host, *Lymnaea truncatula.* Known for its amphibious nature, this snail species thrives in such environments, thereby contributing to the higher prevalence of fasciola in the area.

The prevalence of Paramphistomum in the current study was 11.7%. Notably, a higher prevalence was observed in cattle (15.7%), followed by sheep (7.7%) and goats (3.6%). This is similar to previous studies reported by (24,45,46), which also reported bovine Paramphistomum as being more common. Interestingly, Batu had a high prevalence of Paramphistomum infection (17.5%) compared to Asela and Hawassa study areas. The ecological characteristics of the area such as lowland and presence of lake may have contributed to this heightened occurrence of paramphistomes. The presence of Batu (Ziway) Lake in this vicinity could provide a suitable habitat for the survival of the intermediate host, aquatic snails. Paramphistomes uses diverse range of freshwater snail vectors, including *Bulinus, Biomphalaria, Ceratophallus spp*., and play a role in the transmission of trematodes (47).

The overall prevalence of Schistosoma in our study (6.3%) was found to be lower than that previously reported by (48), (25), (49), (50), (51), and (52). In terms of its suitable agro-ecology, a significant proportion of the study animals, approximately 12.5% (95% CI: 8.3% to 17.9%), were found to have Schistosoma infections in the Batu area, which is characterized by lowland area. Lack of Schistosoma detection in the Asela area is in line with our expectations; but, the result from Hawassa is contrary to what we anticipated. Several factors may account for the observed variations across the studies, including differences in study areas, climatic and ecological variations among study sites, and variations in animal husbandry practices. Additionally, Schistosoma eggs were not detected in Asela and Hawassa, which affect the overall prevalence. In contrast to previous reports by (50,51), no significant differences were found between the prevalence of ovine (3.6%) and bovine (3.5%). The prevalence of Schistosoma species in our study showed no significant difference between ovine (3.6%) and bovine (3.5%) infections, which contrasts with the findings of some earlier studies (50). As grazing animals, both sheep and cattle are likely to have similar exposure and susceptibility to trematode infections if the prevailing Schistosoma species is not so species-specific.

Out of a total of 520 animals slaughtered and underwent examination for trematodes, 108 and 118 of them were infected by Fasciola and Paramphistomum, resulting in prevalence of 20.5%, 22.7%, respectively. The relatively high occurrence of paramphistomum could be partly attributed to the limited availability of effective drugs for its treatment. Common anthelmintic (e.g. Albendazole), used for regular deworming against significant nematodes and liver flukes in Ethiopia have little to no effect on paramphistomes (53). The biology of the parasite and the highly prolific nature of the intermediate host could also contribute to increased prevalence of the parasite (35).

In contrast to what was anticipated and reported in previous studies (52,54–56), our abattoir survey found no evidence of schistosoma prevalence in our research. This could be related to the use of less sensitive diagnostic methods, such as visual inspection of veins, and variations in the geographic origins of the study animals, that include regions where Schistosoma is not prevalent.

In the present study, the prevalence of fasciola showed higher infection rates in Asela (45.8%) compared to Hawassa (18.5%) and Batu (11.3%), indicating distinct agro-ecological trends. This increased occurrence of fasciola along the altitude gradient suggests increasing altitude creates suitable conditions favoring its intermediate host. In highland agro-ecologies, where there is typically cooler temperatures and higher rainfall, fasciolosis due to *F. hepatica* tends to be more prevalent among ruminant populations (57). The highland environmental conditions, characterized by abundant moisture and suitable temperatures, create optimal habitats for the intermediate hosts of Fasciola hepatica, primarily freshwater snails *Lymnae truncatula* which were also abundant in Asela area. Indeed, these snails exhibit amphibious nature, often found in diverse aquatic habitats. They inhabit shallow water bodies, such as ponds, streams, and slow-moving rivers, as well as terrestrial ecology such as muddy substrates. (58) forecasted the varying degrees of *F. hepatica* risk across the country, particularly in wet and humid areas. Their projections suggest that the central highlands, such as Asela, will experience the highest occurrence of *F. hepatica* during the major rainy season (July to September), which provide favorable conditions for snail proliferation and the consequent transmission of the parasite to grazing animals.

In our post-mortem examination, a higher prevalence of paramphistomum was observed in bovine (24.9%) than small ruminants (15.1%), which agrees with the prevalence reported in previous studies. Paramphistomum prevalence figures vary widely across different regions of Ethiopia, as documented by various researchers (14). For instance, (46) reported prevalence of 40.1% in cattle, 28.9% in sheep, and 16.5% in goats from Bishoftu area. Similarly, (45) found prevalence of 65.3% in cattle and 23.7% in sheep from Ashenge area of Tigray region. Occurrences of paramphistomum among ruminants have been observed in various parts of the country, with approximately 45.83% prevalence in western Gojam, 28.6% in Bishoftu, and 6.7% in Hawassa areas. Notably, higher prevalence figures are often observed in cattle compared to small ruminants (14). Cattle graze closer to water bodies and on flat landscape besides being less selective and bite larger volume compared to sheep and goats (54). As a result, they are more likely to consume vegetation and water contaminated with larvae, which increases their exposure to trematode infections (59,60). Cattle also possess a larger rumen capacity in comparison to small ruminants, enabling them to ingest larger volumes of contaminated forage, which likely increase the likelihood of ingesting infective larvae.

Our study identified a significant association between the prevalence of trematodes and the age of animals, both in fecal examination and in the abattoir survey. Similar findings were reported by other authors including (46), (61), (41), (62), and (63). As animals age, their likelihood of exposure to fasciola and paramphistomum larvae increases due to prolonged grazing exposure and higher intake of forage compared to young animals.

Furthermore, the coprological examination demonstrated that the prevalence of Fasciola was higher in non-dewormed animals and lower in the dewormed animals. This underscores the effectiveness of deworming in reducing parasite burden in ruminants and minimizing pasture contamination (64). Conversely, this study revealed an association between prevalence of Fasciolosis and body condition. Consistent with other findings(65) and (66), prevalence of fasciolosis was higher in poorly conditioned animals compared to those in good body condition. This suggests that there is a positive co-relation between animals with poor body condition and parasite infection. The possible predisposing factors may include malnutrition or other health issues, which makes them more susceptible to infections caused by parasites.

In our current investigation, we collected specimens from the study areas and snails belonging to three genera were identified, a result consistent with a previous study (67). In contrast to the findings from another study by (68), our study areas exhibited a higher abundance of *Lymnaea spp*, followed by *Biomphalaria spp* and *Bulinus spp*. Lymnaea, a freshwater snail, serves as the intermediate host for *Fasciola*. Therefore, the observed higher prevalence of *Fasciola* infections in our study may be attributed to the large population of Lymnaeid snails in the study area. Conversely, our study revealed that the distribution of Biomphalaria and Bulinus species is restricted to Batu and Hawassa. This finding aligns with two previous studies (69)and (70), which suggest that the aquatic habitat and ecology of the Ethiopian Rift Valley favor these snail vectors.

**5. CONCLUSION**

We carried out a comprehensive investigation, analyzing 784 ruminant fecal samples and 520 abattoir samples alongside collection and identification of snail vectors. Considerable proportions of ruminants were infected with Fasciola, Paramphistomum, and Schistosoma species. Fasciola was notably more prevalent in Asela compared to Batu and Hawassa. Similarly, Paramphistomum showed a higher prevalence in animals from Batu, while Schistosoma eggs were exclusively found in Batu, characterized by a lowland agro-ecology. Significant associations were noted between trematode infections and various risk factors, including agro-ecology, animal species, body condition score, and deworming practices. Moreover, sheep and cattle exhibited significantly higher risk of trematode infection, with incidence rate ratios (IRRs) 2.2 and 2.5 times more compared to goats, respectively. Furthermore, the identification of snail vector species provided valuable insights into the distribution of potential intermediate hosts and the epidemiology of trematodes infecting livestock. Overall, our findings emphasize the need for effective control measures to mitigate the impact of trematode infestations on both veterinary and public health in Ethiopia.

**Acknowledgements**

The authors extend their gratitude to the Vice President's Office for Research and Technology Transfer of Addis Ababa University for the financial support through the Thematic Research Project. We also express our appreciation to the livestock owners for permitting us to sample their animals and to the abattoir workers in the study area for their collaboration during organ inspection. We highly appreciate the technical support and the provision of laboratory facilities at the Veterinary Parasitology laboratories of both Hawassa and Addis Ababa Universities.

**Authors’ contributions**

The authors confirm contribution to the paper as follows: study conception and design by DA, BM, HA and HW; whereas BH, JS, and RK took part in Data collection, BM did analysis and interpretation, all authors including NM, OM, RK and BME played roles in drafting the paper, critical revisions and approval of the final version of the manuscript.

**Ethics declarations**

## Ethics approval and consent to participant

This study received approval from Addis Ababa University and underwent ethical clearance by the Animal Research Ethical Review Committee (Ref. No: VM/ERC/12/02/14/2022). Prior to collecting fecal samples from animals, the objectives and procedures of the study as well as their voluntary participation were thoroughly explained to livestock owners or farmers. Verbal informed consent was obtained from them before proceeding with the study.

**Competing interests:**

The authors declare that they have no competing interests.

**Funding:**

This study was supported by Addis Ababa University Thematic Research on Trematode and Protozoan Diseases (Thematic Project Ref**:** LT/PY-091/2021).

**Availability of data and materials**

All data collected and analyzed during the study is included in this manuscript, and data can be available from the corresponding author on reasonable request.

**6. REFERENCES**

1. Behnke RH, Metaferia F. The contribution of livestock to the Ethiopian economy–Part II. *IGAD LPI Work Pap 02-11* (2011)

2. Kumar N, Rao TKS, Varghese A, Rathor VS. Internal parasite management in grazing livestock. *J Parasit Dis* (2013) 37:151–157.

3. Chai J-Y, Jung B-K. Epidemiology of trematode infections: an update. *Digenetic trematodes* (2019)359–409.

4. Zhang H, Mehmood K. Trematode infection in ruminants, volume II. *Front Vet Sci* (2023) 10:1258751.

5. Bauri RK, Chandra D, Lalrinkima H, Raina OK, Tigga MN, Kaur N. Epidemiological studies on some trematode parasites of ruminants in the snail intermediate hosts in three districts of Uttar Pradesh, Jabalpur and Ranchi. *Indian J Anim Sci* (2015) 85:941–946.

6. de Waal T, Mehmood K. trematode infection in ruminants. *Front Vet Sci* (2021) 8:719577.

7. Kouadio JN, Giovanoli Evack J, Achi LY, Fritsche D, Ouattara M, Silué KD, Bonfoh B, Hattendorf J, Utzinger J, Zinsstag J. Prevalence and distribution of livestock schistosomiasis and fascioliasis in Côte d’Ivoire: results from a cross-sectional survey. *BMC Vet Res* (2020) 16:1–13.

8. Strydom T, Lavan RP, Torres S, Heaney K. The economic impact of parasitism from nematodes, trematodes and ticks on beef cattle production. *Animals* (2023) 13:1599.

9. Kahl A, von Samson-Himmelstjerna G, Krücken J, Ganter M. Chronic wasting due to liver and rumen flukes in sheep. *Animals* (2021) 11:549.

10. Lalor R, Cwiklinski K, Calvani NED, Dorey A, Hamon S, Corrales JL, Dalton JP, De Marco Verissimo C. Pathogenicity and virulence of the liver flukes Fasciola hepatica and Fasciola gigantica that cause the zoonosis Fasciolosis. *Virulence* (2021) 12:2839–2867.

11. Abunna F, Asfaw L, Megersa B, Regassa A. Bovine fasciolosis: coprological, abattoir survey and its economic impact due to liver condemnation at Soddo municipal abattoir, Southern Ethiopia. *Trop Anim Health Prod* (2010) 42:289–292.

12. Abebe R, Abunna F, Berhane M, Mekuria S, Megersa B, Regassa A. Fasciolosis: Prevalence, financial losses due to liver condemnation and evaluation of a simple sedimentation diagnostic technique in cattle slaughtered at Hawassa Municipal abattoir, southern Ethiopia. *Ethiop Vet J* (2010) 14:39–52.

13. Cwiklinski K, O’neill SM, Donnelly S, Dalton JP. A prospective view of animal and human Fasciolosis. *Parasite Immunol* (2016) 38:558–568.

14. Hotessa AS, Kanko DK. Review on paramphistomosis. *Adv Biol Res (Rennes)* (2020) 14:184–192.

15. Leger E, Webster JP. Hybridizations within the genus Schistosoma: implications for evolution, epidemiology and control. *Parasitology* (2017) 144:65–80.

16. Keiser J, Utzinger J. Emerging foodborne trematodiasis. *Emerg Infect Dis* (2005) 11:1507.

17. McManus DP, Dunne DW, Sacko M, Utzinger J, Vennervald BJ, Zhou XN. Schistosomiasis Nat Rev Dis Primers 24: 13. (2018)

18. Dermauw V, Muchai J, Al Kappany Y, Fajardo Castaneda AL, Dorny P. Human fascioliasis in Africa: A systematic review. *PLoS One* (2021) 16:e0261166.

19. Bayu B, Asnake S, Woretaw A, Ali J, Gedefaw M, Fente T, Getachew A, Tsegaye S, Dagne T, Yitayew G. Cases of human fascioliasis in North-West Ethiopia. *Ethiop J Heal Dev* (2005) 19:237–240.

20. Fentie T, Erqou S, Gedefaw M, Desta A. Epidemiology of human fascioliasis and intestinal parasitosis among schoolchildren in Lake Tana Basin, northwest Ethiopia. *Trans R Soc Trop Med Hyg* (2013) 107:480–486.

21. Abaya SW, Mereta ST, Tulu FD, Mekonnen Z, Ayana M, Girma M, Vineer HR, Mor SM, Caminade C, Graham-Brown J. Prevalence of Human and Animal Fasciolosis in Butajira and Gilgel Gibe Health Demographic Surveillance System Sites in Ethiopia. *Trop Med Infect Dis* (2023) 8:1–16. doi: 10.3390/tropicalmed8040208

22. Mehmood K, Zhang H, Sabir AJ, Abbas RZ, Ijaz M, Durrani AZ, Saleem MH, Rehman MU, Iqbal MK, Wang Y. A review on epidemiology, global prevalence and economical losses of fasciolosis in ruminants. *Microb Pathog* (2017) 109:253–262.

23. Regasa A, Seboka M. Review on Fasciolosis, its effect on meat quality/hazards and economical importances. *Entomol Ornithol Herpetol* (2021) 10:245.

24. Yeneneh A, Kebede H, Fentahun T, Chanie M. Prevalence of cattle flukes infection at Andassa Livestock Research Center in north-west of Ethiopia. *Veterinary Research Forum*. Faculty of Veterinary Medicine, Urmia University, Urmia, Iran (2012). p. 85

25. Aragaw K, Tilahun H. Coprological study of trematode infections and associated host risk factors in cattle during the dry season in and around Bahir Dar, northwest Ethiopia. *Vet Anim Sci* (2019) 7:100041. doi: 10.1016/j.vas.2018.11.002

26. Turuna G, Adugna B. Prevalence of Major Bovine Trematodes (Fasciola and Paramphistomum) in Cattle Slaughtered at Nekemte Municipal Abattoir, East Wollega, Oromia Regional State, Ethiopia. *Prevalence* (2019) 9:

27. Yifat D, Kelay B, Bekana M, Lobago F, Gustafsson H, Kindahl H. Study on reproductive performance of crossbred dairy cattle under smallholder conditions in and around Zeway, Ethiopia. *Parity* (2012) 277:

28. Hordofa D, Abunna F, Megersa B, Abebe R. Incidence of morbidity and mortality in calves from birth to six months of age and associated risk factors on dairy farms in Hawassa city, southern Ethiopia. *Heliyon* (2021) 7:e08546.

29. Endale S, Verschuur M, Baars R, Tesfahun B, Misganaw G, Haile D. Opportunities for Scaling up Climate-Smart Dairy Production in Ziway-Hawassa Milk Shed, Ethiopia. *ESAP P* (2021)53.

30. Zeryehun T, Alemu B. Major gross lesions of lung in cattle slaughtered at hawassa municipal abattoir, Southern Ethiopia. *J Vet Med* (2017) 2017:

31. Thrusfield M. *Veterinary epidemiology*. John Wiley & Sons (2018).

32. Jilo SA, Abadura SZ, Adem ME, Fereda AD, Kibi BD, Alemu HH, Ayele ST, Hussein SA, Gelchu AA, Usmael B. Epidemiology of Fasciolosis in cattle in selected districts of bale zone, south eastern Ethiopia. *Prevalence* (2021) 400:48.

33. Nicholson MJ, Butterworth MH. *A guide to condition scoring of zebu cattle*. ILRI (aka ILCA and ILRAD) (1986).

34. Lahunta A de, Habel RE. *Applied veterinary anatomy.* (1986).

35. Urquhart G, Armour J, Duncan J, Dunn A, Jennings F. Veterinary Parasitology 2nd Edition. University of Oxford. (1996)

36. Hansen J, Perry B. The epidemiology, diagnosis and control of helminth parasites of ruminants. (1994)

37. Organization WH. *Bench aids for the diagnosis of intestinal parasites*. World Health Organization (2019).

38. Brown DS. *Freshwater snails of Africa and their medical importance*. CRC press (1994).

39. ITAGAKI H, SUZUKI N, ITO Y, HARA T, WONDE T. Study on the Ethiopian freshwater molluscs, especially on identification, distribution and ecology of vector snails of human schistosomiasis. *Japanese J Trop Med Hyg* (1975) 3:107–134.

40. Mandahl-Barth G. Key to the identification of East and Central African freshwater snails of medical and veterinary importance. *Bull World Health Organ* (1962) 27:135.

41. Kebede IA, Beriso TE, Mengistu TS. Study on Cattle Trematodiasis and Related Risk Factors in Damot Sore District , Wolaita Zone , Southern Ethiopia. (2023) 2023:

42. Ayelign M, Alemneh T. Study on prevalence and economic importance of bovine fasciolosis in three districts of North-East Amhara Region, Ethiopia. *J Infect Non Infect Dis* (2017) 3:24.

43. Asrede T, Shifaw A. Coprological Study on the Prevalence of Ovine Fasciolosis in Debre Birhan Agricultural Research Center, Ethiopia. *Eur J Biol Sci* (2015) 7:103–107.

44. Regasa A, Mesfin B, Adem M. Study on Prevalence and Risk Factors of Ovine Fasciolosis on Shirka District, Arsi Zone, Eastern Ethiopia. *J Vet Med Heal* (2022) 6:2.

45. Kifleyohannes T, Kebede E, Hagos Y, Weldu K, Michael MG. Prevalence of paramphistomosis in ruminants in Ashenge, Tigray Ethiopia. *Acta Parasitol Glob* (2015) 6:83–86.

46. Sintayehu M, Mekonnen A. Prevalence and intensity of Paramphistomum in ruminants slaughtered at Debre Zeit industrial abattoir, Ethiopia. *Glob Vet* (2012) 8:315–319.

47. Ates C, Umur S. Paramphistome species in water buffaloes and intermediate hosts in the Kızılırmak Delta in Samsun Province, Turkey. *Acta Parasitol* (2021) 66:213–221.

48. Almaz H, Tamiru N, Asegedech S. Pathology of natural infections of Schistosoma bovis in cattle in Ethiopia, Bahir Dar, Ethiopia. *Glob Vet* (2013) 11:243–247.

49. Chanie M, Dejen B, Fentahun T. Prevalence of cattle schistosomiasis and associated risk factors in Fogera cattle, South Gondar zone, Amhara National Regional State, Ethiopia. *J Adv Vet Res* (2012) 2:153–156.

50. Kerie Y, Seyoum Z. Bovine and ovine schistosomiasis: Prevalence and associated host factors in selected sites of South Achefer district, northwest Ethiopia. *Thai J Vet Med* (2016) 46:561–567. doi: 10.56808/2985-1130.2775

51. Merawe M, Amssalu YHK, Afera B. Intestinal schistosomiasis of bovine and ovine in Fogera district, South gonder zone, Amhara national regional state, Ethiopia. *Young* (2014) 94:9–57.

52. Lulie B, Guadu T. Bovine schistosomiasis: A threat in public health perspective in Bahir Dar town, northwest Ethiopia. *Acta Parasitol Glob* (2014) 5:1–6.

53. Rolfe PF, Boray JC. Chemotherapy of paramphistornosis in sheep. *Aust Vet J* (1988) 65:148–150.

54. Gebru A, Asaye M, Alemneh T. The Prevalence of Bovine Shistosomiasis in Dembia Woreda, Ethiopia. *Acta Parasitol Glob* (2015) 6:112–116.

55. Molla G, Tintagu T, Yasin A, Alemu B, Assen AA, Tadesse K. Bovine schistosomiasis in some selected areas of South wollo and oromia zones of Amhara region, north-east Ethiopia. *PLoS One* (2022) 17:e0259787.

56. Sumanth S, D’Souza PE, Jagannath MS. A study of nasal and visceral schistosomosis in cattle slaughtered at an abattoir in Bangalore, South India. *Rev Sci Tech Int des Epizoot* (2004) 23:937–942.

57. Ayele M, Hiko A. Review on the Biology of Fasciola Parasites and the Epidemiology on Small Ruminants. *Adv Life Sci Technol* (2016) 48:19–27.

58. Yilma JM, Malone JB. A geographic information system forecast model for strategic control of fasciolosis in Ethiopia. *Vet Parasitol* (1998) 78:103–127.

59. Cuchillo‐Hilario M, Wrage‐Mönnig N, Isselstein J. Forage selectivity by cattle and sheep co‐grazing swards differing in plant species diversity. *Grass Forage Sci* (2018) 73:320–329.

60. Boval M, Sauvant D. Ingestive behaviour of grazing ruminants: Meta-analysis of the components linking bite mass to daily intake. *Anim Feed Sci Technol* (2021) 278:115014.

61. Belete K. A cross sectional study on the coprological prevalence of ovine fasciolosis in Amhara Sayint District, Ethiopia. *J Vet Med Res* (2017)

62. Zewde A, Bayu Y, Wondimu A. Prevalence of Bovine Fasciolosis and Its Economic Loss due to Liver Condemnation at Wolaita Sodo Municipal Abattair, Ethiopia. *Vet Med Int* (2019) 2019: doi: 10.1155/2019/9572373

63. Rizwan HM, Usman M, Naeem MA, Farid MU, Younus M, Sajid MS, Tahir UB, Luqman N, Abbas H, Ateeq MK. Prevalence of ruminant paramphistomosis and comparative histopathology of the infected rumens in Narowal district, Punjab, Pakistan. *Helminthologia* (2022) 59:377–384.

64. Kustritz MR. Parasite Control. *Vet Prev Med* (2022)

65. Kassie BS, Ali SF. Study on the Prevalence and Economic Significance of Bovine Fasciolosis at Gondar Elfora Abattoir, Gondar, Ethiopia. *J Anim Res* (2019) 9:623–631.

66. Mathewos M, Endale H, Kebamo M. Coprological and postmortem assessment and economic significance of bovine fasciolosis in cattle slaughtered at Tarcha Municipal Abattoir, Southern Ethiopia. *Parasite Epidemiol Control* (2023) 22:e00316.

67. Yusuf Z, Dagne K, Erko B, Siemuri O. Polyploidy in Bulinid Snails, with emphasis on Bulinus truncatus/tropicus complex (Planorbidae: pulmonate mollusks) from various localities in Ethiopia. *World J Cell Biol Gen* (2017) 3:11–20.

68. Olkeba BK, Boets P, Mereta ST, Yeshigeta M, Akessa GM, Ambelu A, Goethals PLM. Environmental and biotic factors affecting freshwater snail intermediate hosts in the Ethiopian Rift Valley region. *Parasit Vectors* (2020) 13:1–13.

69. Kloos H, Souza C de, Gazzinelli A, Soares Filho BS, Temba P da C, Bethony J, Page K, Grzywacz C, Lewis F, Minchella D. The distribution of Biomphalaria spp. in different habitats in relation to physical, biological, water contact and cognitive factors in a rural area in Minas Gerais, Brazil. *Mem Inst Oswaldo Cruz* (2001) 96:57–66.

70. Mitiku H, Legesse M, Teklemariam Z, Erko B. Transmission of Schistosoma mansoni in Tikur Wuha area, southern Ethiopia. *Ethiop J Heal Dev* (2010) 24:
